# Effective semi-fed-batch saccharification with high lignocellulose loading using co-culture of *Clostridium thermocellum* and *Thermobrachium celere* strain A9

**DOI:** 10.3389/fmicb.2024.1519060

**Published:** 2025-01-07

**Authors:** Sreyneang Nhim, Sirilak Baramee, Chakrit Tachaapaikoon, Patthra Pason, Khanok Ratanakhanokchai, Ayaka Uke, Ruben Michael Ceballos, Akihiko Kosugi, Rattiya Waeonukul

**Affiliations:** ^1^Enzyme Technology Laboratory, School of Bioresources and Technology, King Mongkut’s University of Technology Thonburi (KMUTT), Bangkok, Thailand; ^2^Excellent Center of Enzyme Technology and Microbial Utilization, Pilot Plant Development and Training Institute (PDTI), King Mongkut’s University of Technology Thonburi (KMUTT), Bangkok, Thailand; ^3^Biological Resources and Post-harvest Division, Japan International Research Center for Agricultural Sciences (JIRCAS), Ibaraki, Japan; ^4^Molecular and Cell Biology Department, School of Natural Sciences, University of California, Merced, Merced, CA, United States; ^5^Center of Excellence on Petrochemical and Materials Technology, Chulalongkorn University, Bangkok, Thailand

**Keywords:** *Clostridium thermocellum*, co-culture, lignocellulose, saccharification, semi-fed-batch, *Thermobrachium celere*

## Abstract

Maximizing saccharification efficiency of lignocellulose and minimizing the production costs associated with enzyme requirements are crucial for sustainable biofuel production. This study presents a novel semi-fed-batch saccharification method that uses a co-culture of *Clostridium thermocellum* and *Thermobrachium celere* strain A9 to efficiently break down high solid-loading lignocellulosic biomass without the need for any external enzymes. This method optimizes saccharification efficiency and enhances glucose production from alkaline-treated rice straw, a representative lignocellulosic biomass. Initially, a co-culture of *C. thermocellum* and *T. celere* strain A9 was established with a treated rice straw loading of 150 g/l, supplemented with Tween 20, which enhanced enzymes stability and prevented unproductive binding to lignin, achieving a remarkable glucose concentration of up to 90.8 g/l. Subsequently, an additional 100 g/l of treated rice straw was introduced, resulting in a total glucose concentration of up to 140 g/l, representing 70.1% of the theoretical glucose yield from the 250 g/l treated rice straw load. In contrast, batch saccharification using an initial substrate concentration of 250 g/l of alkaline-treated rice straw without Tween 20 resulted in a glucose concentration of 55.5 g/l, with a theoretical glucose yield of only 27.7%. These results suggest that the semi-fed-batch saccharification method using co-cultivation of *C. thermocellum* and *T. celere* strain A9, supplemented with Tween 20 is an efficient microbial method for saccharifying high-concentration biomass. Moreover, this approach effectively manages high solids loading, optimizes efficiency, and reduces the need for external enzymes, thus lowering production costs and simplifying the process for industrial applications.

## 1 Introduction

The increasing demand for sustainable energy has propelled research into biofuels derived from renewable resources (Neupane, 2022; Lopez et al., 2023). Lignocellulosic biomass, comprised of cellulose, hemicellulose, and lignin, is abundant and renewable (Ceballos, 2017; Zheng et al., 2022). Therefore, it has high potential as a sustainable feedstock for bioethanol production (Vasić et al., 2021; Broda et al., 2022; Jahangeer et al., 2024). However, the biofuel industry faces critical challenges in achieving economically viable processes for lignocellulose-to-bioethanol conversion. These challenges include the high cost of enzymatic hydrolysis and the scalability of current methods to industrial applications, both of which hinder broader adoption in the energy market.

Efficient conversion of lignocellulosic biomass into fermentable sugars remains challenging, primarily due to the recalcitrant nature of lignocellulose and lignin in particular (Agrawal et al., 2021; Broda et al., 2022; Saini et al., 2022). Effective saccharification of lignocellulose requires robust enzyme systems capable of deconstructing complex carbohydrates into simple sugars (Guo et al., 2018; Østby et al., 2020). In addition to degrading or otherwise removing lignin, another critical aspect of improving lignocellulosic biomass conversion is maximizing the loading of solid substrate during saccharification. This can significantly boost sugar yields, thus enhancing downstream fermentation efficiency (Modenbach and Nokes, 2013; Shiva et al., 2022). However, high solid loading poses challenges such as increased viscosity and mass transfer limitations, which in turn reduces enzyme access to substrate and thus hydrolytic efficiency (Jung et al., 2015; da Silva et al., 2020). To resolve these issues, a variety of strategies are being explored, including optimizing enzyme cocktails, employing enzyme platforms, adopting fed-batch approaches, and incorporating chemical additives such as surfactants (Mitsuzawa et al., 2009; Ceballos et al., 2014; Gomes et al., 2018; Guo et al., 2018; Mukasekuru et al., 2020; Østby et al., 2020; Saini et al., 2022).

Among these strategies, non-ionic surfactants like Tween 20 have demonstrated significant potential in enhancing lignocellulose saccharification efficiency (Eriksson et al., 2002; Seo et al., 2011; Eckard et al., 2013; Chen et al., 2018; Cui et al., 2023). Prior studies have elucidated several mechanisms through which Tween 20 improves this process. For instance, Seo et al. (2011) examined the effect of Tween 20 on the enzymatic hydrolysis of pine wood chips with varying lignin contents and found that its primary role was to increase the effective accessible surface area of cellulose for cellulase binding. Similarly, Chen et al. (2018) found that Tween 20 reduced protein adsorption on acid-pretreated wheat straw, thereby enhancing enzymatic glucan conversion. This effect was attributed to Tween 20’s ability to block lignin-cellulase interactions and modify lignin surface properties, such as hydrophobicity, hydrogen bonding, and surface charges, to reduce non-productive protein adsorption. Wang et al. (2020) explored the impact of Tween 20 on enzymatic hydrolysis and enzyme adsorption in pretreated lignocellulosic materials. Their findings indicated that the improvement in hydrolysis was not solely due to blocking cellulase adsorption on lignin but was more related to structural changes in the lignocellulosic material, such as hemicellulose removal and surface morphology modifications. Additionally, Tiwari et al. (2022) reported that alkali pretreatment of sugarcane bagasse, combined with the addition of Tween 20 during enzymatic hydrolysis, synergistically improved saccharification yield by increasing the substrate’s surface area. Furthermore, several studies have shown that Tween 20 enhances enzymatic hydrolysis rates under high solid-loading conditions by reducing slurry viscosity and improving substrate-enzyme interactions (Seo et al., 2011; Liu et al., 2016; Chen et al., 2018; Wang et al., 2020; Cui et al., 2023). This improvement not only increases saccharification efficiency but also reduces enzyme requirements per batch, ultimately lowering production costs.

In conventional enzymatic hydrolysis of lignocellulosic biomass, processes often rely on the addition of commercial enzymes, which can significantly increase production costs (Ceballos, 2017). Furthermore, enzyme production for lignocellulose hydrolysis is constrained due to the use of expensive synthetic media and challenges associated with separating cell remnants during the process, making it unsuitable for large-scale biofuel production (Singhania et al., 2021). Additionally, efficient lignocellulose saccharification requires the coordination of multiple enzymes, rather than a single enzyme, which further complicates the process and increases costs (Lv et al., 2022). Consequently, there is considerable interest in developing alternative strategies that can enhance the saccharification process while reducing dependence on externally added enzymes. One approach is to employ microorganisms to directly saccharify lignocellulosic biomass into fermentable sugars (Taha et al., 2015; Du et al., 2020; Beri et al., 2020; Akdemir et al., 2022; Zou et al., 2023). Microbial systems enable simultaneous enzyme production and hydrolysis within a single process, streamlining operations, lowering production costs, and improving overall efficiency. This approach meets the biofuel industry’s demand for scalable, cost-effective solutions and minimizes reliance on high-cost commercial enzymes.

Recent studies have demonstrated the use of microorganisms for the saccharification of lignocellulosic biomass as a promising alternative to traditional enzymatic hydrolysis. Various microorganisms, including bacteria such as *Bacillus* strains (Vu et al., 2022), *Cellulomonas* strain (Ontanon et al., 2021), and *Clostridium thermocellum* (Prawitwong et al., 2013; Nhim et al., 2022); fungi such as *Trichoderma reesei* (Fang et al., 2019) and *Aspergillus niger* (Xue et al., 2020); and engineered yeast such as *Saccharomyces cerevisiae* (Hong et al., 2003) and *Pichia pastoris* (Lu et al., 2015; Kuhad et al., 2016), have been developed to directly convert biomass into fermentable sugars. Among these, bacteria are often preferred for biofuel production from lignocellulose due to their fast growth rates and short generation cycles. Bacterial cellulases are typically bound to the cell membrane, allowing the bacterial cells adsorbed on lignocellulose to perform both enzyme production and saccharification in a single, streamlined process. Thermophilic bacteria, in particular, have gained significant attention for their heat-tolerant enzymes, which are valuable for industrial applications and production processes (Akinosho et al., 2014; Lynd et al., 2017; Du et al., 2020; Liu et al., 2020; Mazzoli and Olson, 2020). Consequently, selecting the appropriate microorganism is essential for efficient lignocellulose saccharification.

Among bacteria, *C. thermocellum* has shown considerable promise due to its optimal growth temperature aligning with that of enzymatic hydrolysis temperature, making it highly suitable for microbial saccharification (Akinosho et al., 2014; Lynd et al., 2017; Du et al., 2020; Liu et al., 2020; Mazzoli and Olson, 2020). Also known as *Ruminiclostridium thermocellum*, *Hungateiclostridium thermocellum*, or *Acetivibrio thermocellus*, *C. thermocellum* is an anaerobic thermophilic bacterium recognized for its efficient degradation of cellulosic biomass using multienzyme complexes, called cellulosomes (Lynd et al., 2002; Bayer et al., 2004). Cellulosome architecture allows a diverse array of enzymes to be selectively activated for breaking down cellulosic biomass (Bayer et al., 2004). However, absence of *β*-glucosidases, is a major limitation of the *C. thermocellum* cellulosome. The lack of β-glucosidase induces feedback inhibition through cellobiose accumulation (Lamed et al., 1991; Tachaapaikoon et al., 2012; Waeonukul et al., 2012).

One strategy to overcome this limitation includes the screening natural or engineered *β*-glucosidases for enhanced hydrolytic capacity on cellobiose in saccharification systems (Gefen et al., 2012; Waeonukul et al., 2012; Hirano et al., 2019; Qi et al., 2021). Prior results from our research showed that *C. thermocellum* cultures supplemented with thermostable *β*-glucosidase resolves cellobiose feedback inhibition and improves saccharification (Prawitwong et al., 2013). This approach called biological simultaneous enzyme production and saccharification (BSES) aligns with results from other groups, which have also shown that supplementing cultures of *C. thermocellum* with bacteria that expressed *β*-glucosidase produces significant glucose yields from cellulose (Ichikawa et al., 2019).

Other strategies to overcome cellobiose-based feedback inhibition include engineering recombinant *C. thermocellum* strains that produce hybrid enzymes integrated into the cellulosome via a dockerin module. One such strain is *Caldicellulosiruptor* sp. F32, which expresses CelS linked to β-glucosidase. This approach enhances glucose production from microcrystalline cellulose substrates by mitigating feedback inhibition (Zhang et al., 2017). However, a drawback in the use of recombinant *C. thermocellum* is optimization of suitable β-glucosidases for integration into the cellulosomes, which can be quite challenging. Biosafety concerns related to the use of genetically modified microbes in industrial settings is another factor impacting industry acceptance of this approach (Friehs, 2004; Kumar, 2014).

Recently, our research demonstrated that *Thermobrachium celere* strain A9, isolated from wastewater sediments, secretes a highly active β-glucosidase into the culture medium. We also demonstrated efficient cellulose saccharification by co-culturing *C. thermocellum* and *T. celere* strain A9. The synergy under anaerobic thermophilic conditions enhanced cellulolytic activity, eliminating the need for exogenous β-glucosidase supplements (Nhim et al., 2022). Thus, we suggested that a co-culture of *C. thermocellum* and *T. celere* strain A9 may be a promising approach for biological lignocellulose saccharification that requires neither supplementation with exogenous β-glucosidase nor recombinant techniques.

In this study, we evaluated the adaptability and efficiency of the *C. thermocellum* and *T. celere* strain A9 co-culture system for saccharifying lignocellulosic biomass (e.g., rice straw) and investigated whether the addition of Tween 20 enhances the saccharification process. Furthermore, we developed a semi-fed-batch saccharification method with high solid loading using and alkali-treated rice straw as a representative substrate. Through this method, we aim to enhance saccharification efficiency and reduce the need for additional enzyme supplements. Our results suggest that semi-fed-batch saccharification using this co-culture strategy is effective and economical, with the potential to simplify the bioconversion of lignocellulosic biomass and better align with the needs of the biofuel industry.

## 2 Materials and methods

### 2.1 Organisms, media, and growth conditions

*C. thermocellum* ATCC 27405 was obtained from the American Type Culture Collection (ATCC) and cultivated in BM7CO medium at pH 7.0, which contained 10 g/l of microcrystalline cellulose (Sigmacell type 20; Sigma-Aldrich, St. Louis, MO, United States). *T. celere* strain A9, deposited with the National Institute of Technology and Evaluation (NITE) as NITE P-03545, was also grown in BM7CO at pH 7.0, but with 5 g/l of cellobiose (Sigma-Aldrich, St. Louis, MO, USA). The composition and preparation of the BM7CO medium were carried out as described by Nhim et al. (2022).

*S. cerevisiae* TISTR 5019, which was obtained from the Thailand Institute of Scientific and Technological Research (TISTR) Culture Collection Center (Pathum Thani, Thailand), exhibits high tolerance to both glucose and ethanol. For culturing, *S. cerevisiae* was grown aerobically in static culture at 30°C on complete medium (YPD) comprising 20 g/l peptone, 10 g/l yeast extract (Difco Laboratories, Detroit, MI, United States), and 20 g/l glucose.

### 2.2 Preparation of alkali-pretreated lignocellulose biomass and compositional analysis

Lignocellulosic biomass samples, including corn hulls, rice straw, oil palm empty fruit bunches, and sugarcane bagasse, were sourced from various locations in Thailand. Each biomass type was ground to a fine particle size using a 0.5 mm mesh screen (ZM-100; Retsch, Haan, Germany) and pretreated using the alkaline pretreatment method described by Prawitwong et al. (2013).

The chemical composition of both natural and alkaline-treated lignocellulosic biomass was analyzed in accordance with the National Renewable Energy Laboratory (NREL) standard procedures.^1^ The sugar composition of acid-hydrolyzed biomass was determined using high-performance liquid chromatography (HPLC) (Shimadzu Corp., Kyoto, Japan) with an Aminex HPX-87H column (Bio-Rad, Hercules, CA, United States). Sulfuric acid (1 mM) was used for the mobile phase with a flow rate of 0.6 ml/min detected on a Prominence instrument (Shimadzu Corp., Kyoto, Japan) operated at 60°C and equipped with a Shimadzu RID-10A refractive index detector. Acid-insoluble lignin (Klason lignin) was quantified by weighing the filter cake after drying it at 70°C to a constant weight.

### 2.3 Enzyme and protein assays

Cellulase activity was measured by determining the amount of reducing sugars released from microcrystalline cellulose. The reaction mixture consisted of 0.5% (w/v) microcrystalline cellulose in 100 mM sodium acetate buffer (pH 6.0) and was incubated at 60°C for 30 min. The increase in reducing sugars was quantified using the Somogyi-Nelson method (Somogyi and Nelson, 1944), with glucose as the standard. One unit of cellulase activity was defined as the amount of enzyme required to release 1 μmol of glucose equivalent per minute under the assay conditions.

*β*-Glucosidase activity was determined by measuring the hydrolysis of *p*-nitrophenol-β-D-glucoside (*p*NPG; Sigma-Aldrich, St. Louis, MO, United States), as described by Waeonukul et al. (2012). One unit (U) of β-glucosidase activity was defined as the amount of enzyme that liberated 1 μmol of *p*-nitrophenol per minute per milliliter.

Protein concentrations were determined using a Coomassie protein assay kit (Thermo Fisher Scientific, Waltham, MA, United States) with bovine serum albumin as the standard.

### 2.4 Saccharification of lignocellulose by *C. thermocellum* and *T. celere* strain A9 co-culture

The saccharification process by co-culture of *C. thermocellum* and *T. celere* strain A9 was adapted from previous work (Nhim et al., 2022). For sub-culturing, *C. thermocellum* ATCC 27405 stock culture was inoculated into 5 ml of BM7CO medium containing 10 g/l cellulose and incubated anaerobically at 60°C. The subculture of *C. thermocellum* ATCC 27405 was inoculated again into BM7CO medium containing 50 g/l alkali-pretreated lignocellulosic biomass for 2 days. A subculture of *T. celere* strain A9 was inoculated into *C. thermocellum* cultures after a 2-day cultivation of the latter. A 10% (v/v) inoculum was used for both strains. Then the co-cultures of *C. thermocellum* and *T. celere* strain A9 were incubated anaerobically at 60°C for an additional 7 days. Glucose accumulation in culture supernatant was continuously monitored by HPLC. Glucose yield (%) was calculated based on the amount of glucose released relative to the dry weight of lignocellulosic biomass. All experiments were performed in triplicate.

### 2.5 Effect of inoculation timing of *T. celere* strain A9 on saccharification using co-culture

The effect of inoculation timing of *T. celere* strain A9 into *C. thermocellum* cultures on the saccharification of lignocellulosic biomass was evaluated using BM7CO medium containing 50 g/l of alkaline-treated rice straw in anaerobic serum bottles. The bottles were sealed, flushed with high-purity CO₂ to establish anaerobic conditions, and autoclaved at 121°C for 20 min. Inoculation strategies included simultaneous inoculation of *C. thermocellum* and *T. celere* strain A9, and staggered inoculations, whereby *C. thermocellum* was cultured first followed by *T. celere* strain A9 inoculation after 1, 2, or 3 days. A 10% (v/v) inoculum size was used for both strains, with incubation at 60°C for 7 days. Daily samples were collected from the co-cultures, and the accumulated glucose in the culture supernatants was quantified using HPLC. All experiments were performed in triplicate.

### 2.6 Effect of solid loading on saccharification using co-culture

The effect of various solid loadings on the saccharification of treated rice straw was evaluated using concentrations of 50, 100, 150, 200, and 250 g/l of the lignocellulosic substrate in BM7CO medium within anaerobic serum bottles. Following the previously described method, *C. thermocellum* and *T. celere* strain A9 were inoculated sequentially, with *C. thermocellum* inoculated first, followed by the addition of *T. celere* strain A9 after 2 days, which was chosen based on the optimal inoculation timing as determined by previous experiments in the study. The co-cultures were then incubated at 60°C for 7 days, and daily samples were collected to monitor saccharification, with glucose concentrations analyzed using HPLC. All experiments were performed in triplicate.

### 2.7 Effect of Tween 20 supplementation on saccharification using co-culture

The effect of Tween 20 supplementation on the saccharification of treated rice straw was evaluated using concentrations of 0.1, 0.5, 1.0, and 1.5% (v/v) in BM7CO medium within anaerobic serum bottles. The concentrations tested (ranging from 0.1 to 1.5% v/v) were chosen to evaluate the optimal range for enhancing saccharification, based on previous studies (Seo et al., 2011; Chen et al., 2018; Wang et al., 2020; Tiwari et al., 2022) and preliminary tests. Sterile Tween 20 was added to the sterile BM7CO medium containing alkaline-treated rice straw to achieve the desired concentrations. These experiments were conducted under optimal conditions determined from early experiments in this present study. Co-cultures were incubated at 60°C for 7 days. Cellulase and *β*-glucosidase activities, as well as the concentration of accumulated glucose in the culture supernatants, were determined using an enzymatic assay and HPLC, respectively. All experiments in this study were performed in triplicate.

### 2.8 Semi-fed-batch biomass saccharification by co-culture supplemented with Tween 20

A semi-fed-batch saccharification process was conducted with optimized conditions identified in prior experiments. Initially, a co-culture of *C. thermocellum* and *T. celere* strain A9 was prepared in BM7CO medium with a solid loading of 150 g/l of treated rice straw, supplemented with 0.5% (v/v) Tween 20. The co-culture was incubated anaerobically at 60°C for 7 days to allow for initial saccharification. Upon completion of this phase, an additional 100 g/l of treated rice straw was incrementally added to the culture to sustain the saccharification process. The culture was then incubated anaerobically at 60°C for an additional 7 days. Cellulase and β-glucosidase activities, as well as the concentration of accumulated glucose in the culture supernatants, were monitored using enzymatic assays and HPLC, respectively. All experiments were performed in triplicate.

### 2.9 Fermentation tests

The semi-fed-batch cultures of *C. thermocellum* and *T. celere* strain A9 were fermented directly through the addition of yeast, without any supplemental additives. The BM7CO medium, which maintained the same glucose concentration, was adjusted to a pH of 7.0 using hydrochloric acid (HCl) and then inoculated with yeast to serve as a reference fermentation profile. *S. cerevisiae* TISTR 5019, previously precultured on YPD medium, was washed twice with sterile water and subsequently inoculated into each medium at a concentration of 5% (v/v). The cultures were incubated at 30°C. Ethanol concentrations in the samples were measured using gas chromatography (GC; Model GC-2014, Shimadzu, Kyoto, Japan) equipped with a flame ionization detector. The analytical column was a DB-WAX (30 m × 0.32 mm × 0.5 μm, Agilent Technologies, Inc., Santa Clara, CA, Unites States). Helium was used as the carrier gas at a constant flow rate of 3.0 ml/min.

### 2.10 Statistical analysis

Data are expressed as the mean ± SD from triplicate experiments. Statistical analyses were performed using IBM SPSS Statistics (SPSS Inc., Chicago, IL, United States). Statistical significance was determined by one-way ANOVA followed by Tukey’s test, with *p* < 0.05 considered statistically significant.

## 3 Results

### 3.1 Selection of lignocellulosic biomass for saccharification by co-culture system

**Figure 1:**
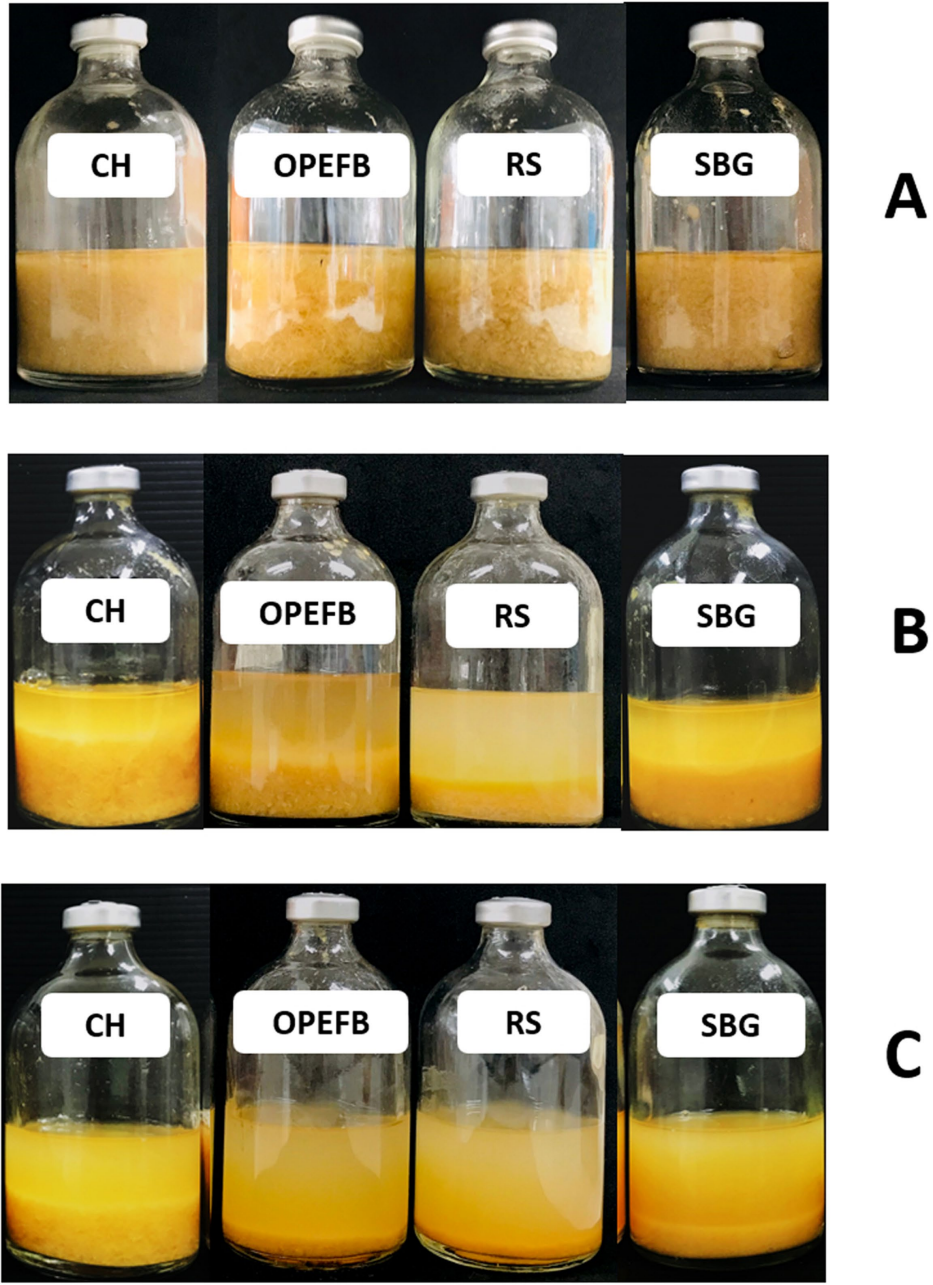
Saccharification of treated lignocellulosic biomass, including corn hull (CH), oil palm empty fruit bunch (OPEFB), rice straw (RS), and sugarcane bagasse (SBG) at a solid loading of 50 g/l, after 7 days. Control without inoculum **(A)**, Monoculture of *C. thermocellum* **(B)**, and Co-culture of *C. thermocellum* and *T. celere* strain A9 **(C)**. Images of the cultivation process are shown for each condition.

**Figure 2:**
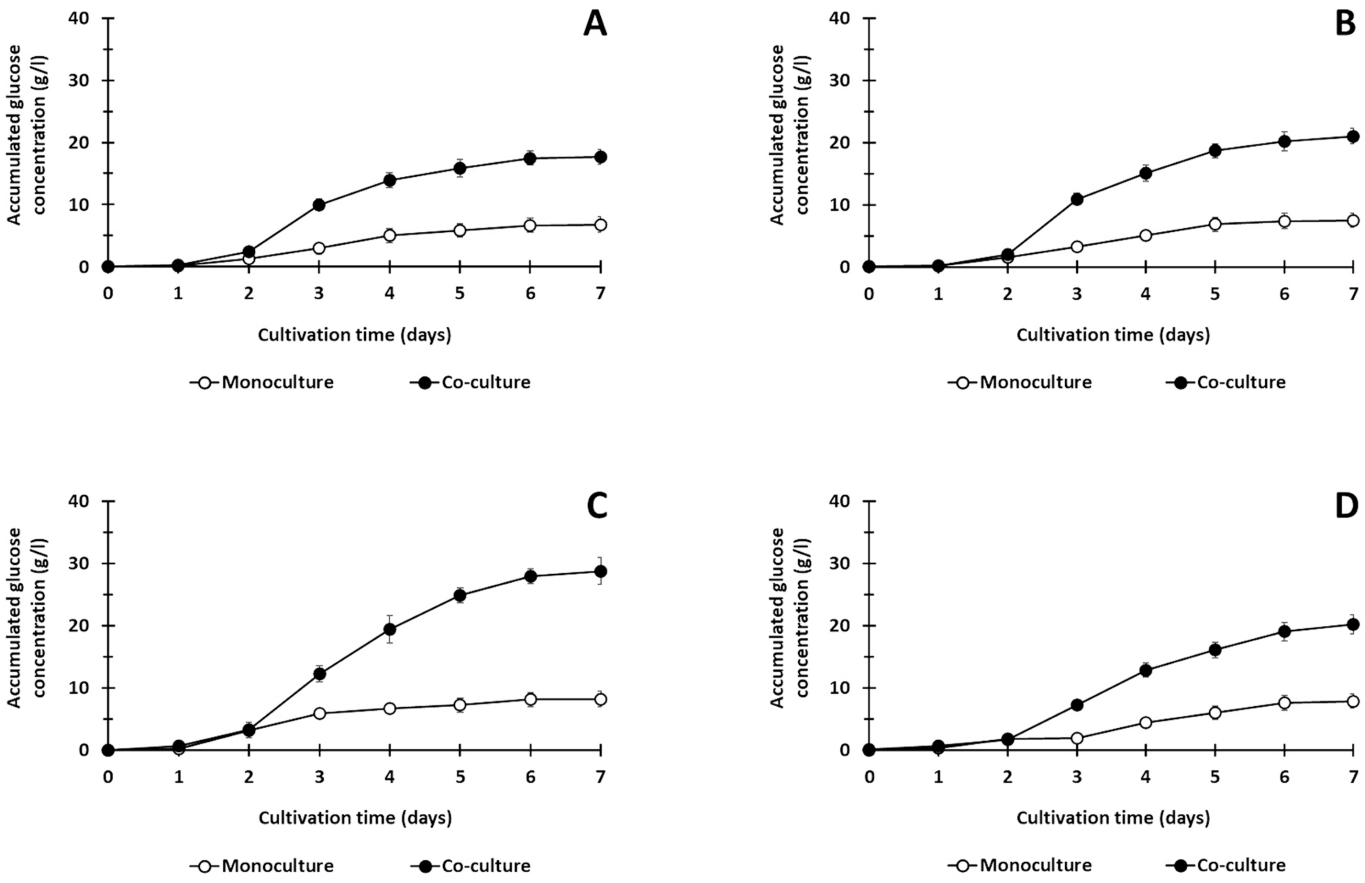
Saccharification of treated lignocellulosic biomass including corn hull **(A)**, oil palm empty fruit bunch **(B)**, rice straw **(C)**, and sugarcane bagasse **(D)** using monoculture of *C. thermocellum* and co-cultures of *C. thermocellum* and *T. celere* strain A9 at a solid loading of 50 g/l. The data are the means of three independent experiments. Error bars represent standard deviations (*n* = 3).

In prior work, we showed that co-culturing *C. thermocellum* and *T. celere* strain A9 efficiently converts cellulose to glucose in BM7CO medium using 50 g/l microcrystalline cellulose as a substrate. Results demonstrated an efficient degradation of cellulose with an accumulation of 35.2 g/l of glucose in the culture supernatant. This represents 70.4% of the maximum theoretical glucose yield (Nhim et al., 2022). In this study, we examined the ability of this co-culture system to degrade lignocellulosic biomass using several complex substrates, including corn hull, oil palm empty fruit bunch, rice straw, and sugarcane bagasse. These substrates were chosen due to their abundance and potential as renewable biomass resources. Lignocellulosic biomass samples were alkaline-pretreated, which has been shown previously by our lab and others to enhance saccharification efficiency (Liu et al., 2018; Das et al., 2021). The chemical composition of alkaline-treated lignocellulosic feedstocks include cellulose, hemicellulose (xylan), and lignin (see Table 1). A concentration of 50 g/l of lignocellulosic substrate was used in these experiments (Figure 1A). Saccharification by *C. thermocellum* monoculture (Figure 1B), yielded net glucose accumulations of 6.8, 7.5, 8.2, and 7.9 g/l from corn hull, oil palm empty fruit bunch, rice straw, and sugarcane bagasse, respectively (Figures 2A–D). Saccharification by co-culture of *C. thermocellum* and *T. celere* strain A9 exhibited significantly enhanced saccharification efficiency (Figure 1C). Specifically, saccharification via co-culture of *C. thermocellum* and *T. celere* strain A9 resulted in noticeable increases in glucose accumulation observed after 2 days of cultivation. Glucose accumulation remained consistent after 7 days of cultivation, indicating sustained saccharification activity (Figures 2A–D). Within this period, glucose accumulation increased to 17.7, 21.0, 28.8, and 20.2 g/l, for corn hull, oil palm empty fruit bunch, rice straw, and sugarcane bagasse, respectively. These yields correspond to 46.6, 55.9, 72.0, and 53.2%, of the theoretical maximum glucose yields, indicating a significant enhancement in yield over *C. thermocellum* monocultures (Figures 2A–D).

**Table 1:** Chemical composition of treated corn hull, oil palm empty fruit bunches, rice straw, and sugarcane bagasse.

Type of pretreated biomass	Composition (% w/w)		
	Cellulose	Xylan	Lignin
Corn hull	75.87 ± 0.48	8.16 ± 0.72	6.61 ± 0.55
Oil palm empty fruit bunch	75.31 ± 0.53	7.70 ± 0.29	8.50 ± 0.31
Rice straw	80.47 ± 0.33	5.30 ± 0.64	6.80 ± 0.36
Sugarcane bagasse	75.75 ± 0.45	8.87 ± 0.37	9.80 ± 0.23

These results indicate that the extracellular *β*-glucosidase of *T. celere* strain A9 cooperates with *C. thermocellum* cellulosomes and effectively resolves cellobiose feedback inhibition. This suggests that *T. celere* strain A9 is effective for biological cellulose saccharification when used in co-culture with *C. thermocellum* without exogenous enzyme supplements. Among the lignocellulosic biomass substrates examined using co-culturing of *C. thermocellum* with *T. celere* strain A9, alkaline-treated rice straw exhibited the highest saccharification efficiency with a conversion rate comparable to that obtained from microcrystalline cellulose (Nhim et al., 2022). Therefore, alkaline-treated rice straw was selected for further investigation.

### 3.2 Optimizing for enhanced saccharification of treated rice straw through co-culture

#### 3.2.1 Effect of inoculation timing of *T. celere* strain A9 on saccharification of treated rice straw

The impact of *T. celere* strain A9 inoculation timing on saccharification of alkaline-treated rice straw using the co-culture system was studied using loading of 50 g/l of treated rice straw. The saccharification efficiency was compared between: strains inoculated simultaneously; and, *C. thermocellum* cultured for 1, 2, and 3 days prior to inoculating with *T. celere* strain A9.

**Figure 3:**
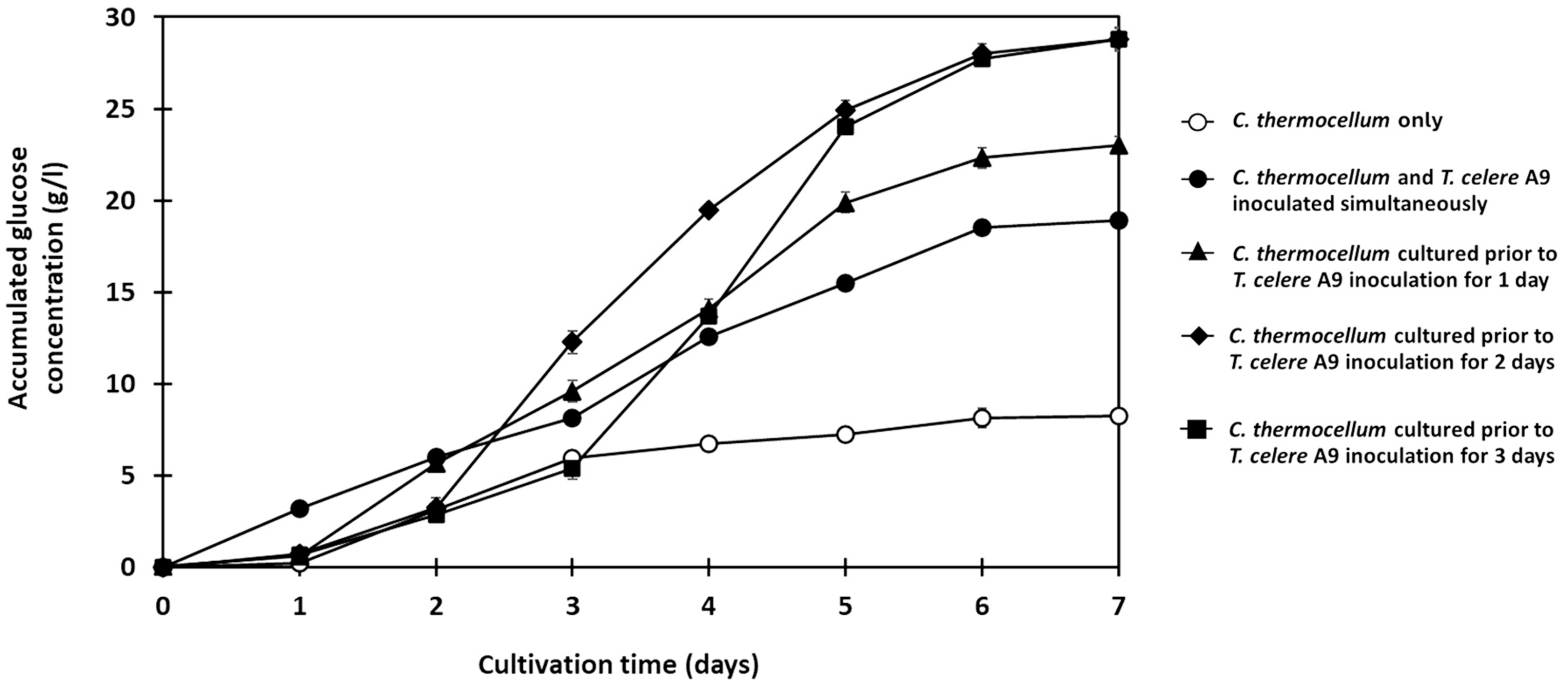
Effect of inoculation timing of *T. celere* strain A9 on the saccharification of treated rice straw at a solid loading of 50 g/l using co-culture with *C. thermocellum*. The data are the means of three independent experiments. Error bars represent standard deviations (*n* = 3).

Results indicate that culturing *C. thermocellum* before adding *T. celere* strain A9 results in higher glucose production over simultaneous inoculation of both strains into culture. In *C. thermocellum* monoculture, only 8.2 g/l of glucose (20.6% of the theoretical glucose yield) was observed (Figure 3). Conversely, when both strains were inoculated simultaneously, glucose production significantly increased to 18.9 g/l (47.4% of the theoretical glucose yield) after 7 days of cultivation (Figure 3).

Longer *C. thermocellum* pre-cultivation periods resulted in increased glucose yields. When *C. thermocellum* was inoculated before adding *T. celere* strain A9 at 1, 2, and 3 days, glucose concentrations in the culture at day 7 were 23.0 g/l, 28.8 g/l, and 28.8 g/l, respectively. This corresponds to 57.5, 72.0, and 72.0% of theoretical yields (Figure 3). No significant difference was observed between the glucose yields for 2 and 3 days of pre-cultivation. Statistical analysis, detailed in Supplementary Table S1, confirmed these trends and differences in glucose production. Therefore, 2 days of *C. thermocellum* pre-cultivation (prior to *T. celere* strain A9 inoculation) was selected for subsequent experiments since this condition maximized glucose yield and no significant improvement was observed for longer pre-cultivation periods.

#### 3.2.2 Effect of solid loading on saccharification of treated rice straw using co-culture

**Figure 4:**
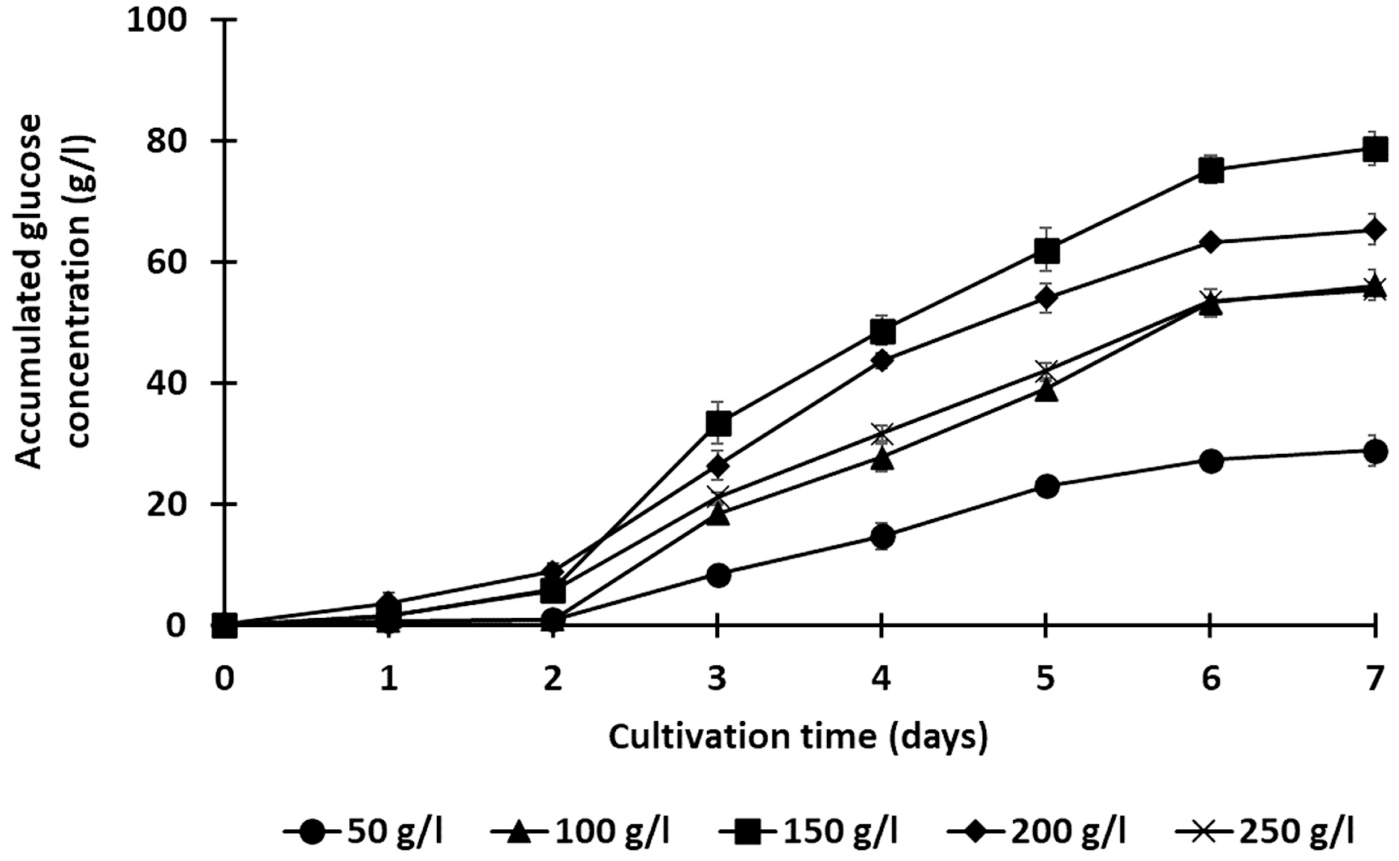
Effect of solid loading (50, 100, 150, 200, and 250 g/l) on the saccharification of treated rice straw using a co-culture of *C. thermocellum* and *T. celere* strain A9. The data are the means of three independent experiments. Error bars represent standard deviations (*n* = 3).

**Figure 5:**
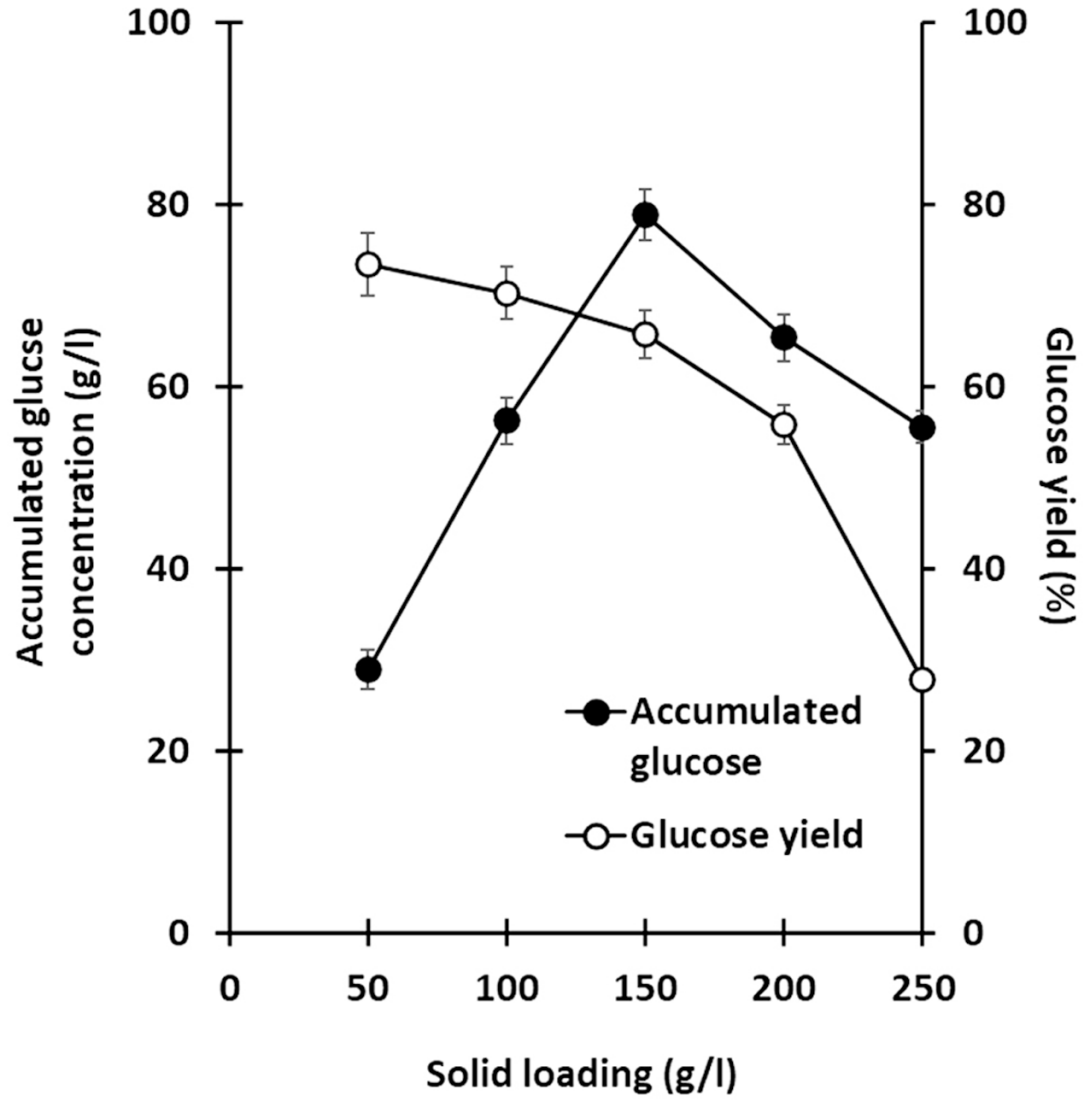
Relationship between accumulated glucose concentration (g/l) and glucose yield (%) at various solid loading. Glucose yield (%) was calculated based on the amount of glucose released relative to the dry weight of treated rice straw. The data are the means of three independent experiments. Error bars represent standard deviations (*n* = 3).

Lignocellulosic substrate deconstruction efficiency was also evaluated under various substrate loading conditions to optimize glucose production using this co-culture system. Solid loadings of alkaline-treated rice straw, ranging from 50 to 250 g/l, were examined, and saccharification performance was monitored over 7 days. In all trials, *C. thermocellum* was cultured for 2 days prior to inoculation with *T. celere* strain A9. Across all solid loading cases, glucose production significantly increased following the inoculation of *T. celere* strain A9 into *C. thermocellum* cultures. Remarkably, the co-culture system maintained efficient glucose production even at the highest substrate loading tested. For substrate loadings ranging from 50 to 250 g/l, glucose concentrations increased with substrate loading up to 150 g/l, reaching 78.8 g/l (65.7% of the theoretical glucose yield). However, glucose concentrations decreased at higher loadings, with a significant drop at 250 g/l (55.5 g/l, or 27.7% of the theoretical glucose yield). This decrease is likely due to the high substrate concentrations exceeding the capacity for bacterial growth and the enzyme system’s ability to efficiently hydrolyze the biomass. The highest glucose concentration was observed at 150 g/l, demonstrating that the co-culture system was most efficient at this solid loading (Figures 4, 5). Supplementary Table S2 provides statistical analysis confirming these trends and significant differences between glucose yields at various solid loadings.

#### 3.2.3 Effect of Tween 20 on saccharification of treated rice straw using co-culture

**Figure 6:**
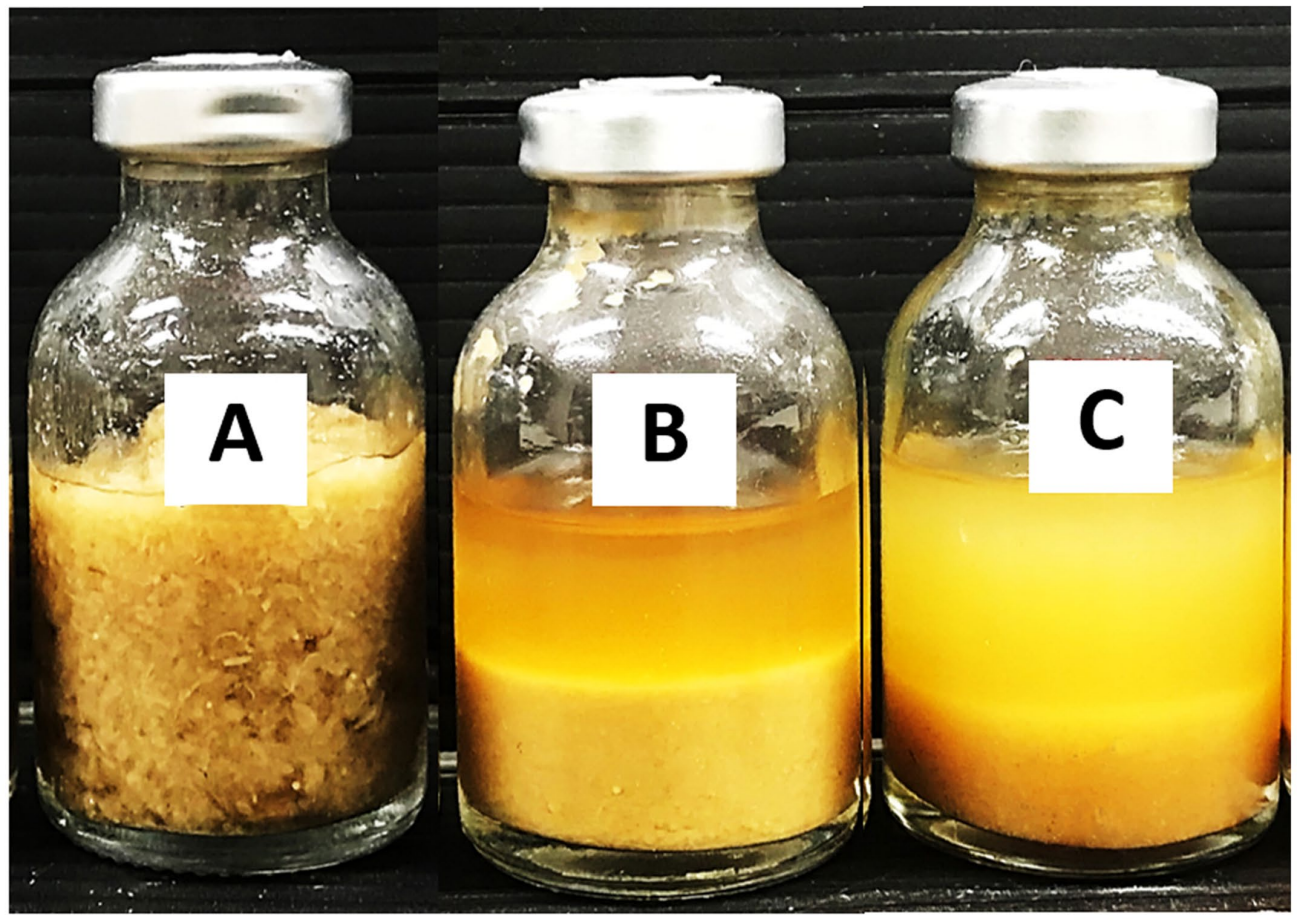
Images showing the effect of Tween 20 supplementation on the saccharification of treated rice straw at a solid loading of 150 g/l using a co-culture of *C. thermocellum* and *T. celere* strain A9. **(A)** Control without inoculum, **(B)** Co-culture without Tween 20 supplementation, and **(C)** Co-culture with 0.5% (v/v) Tween 20 supplementation.

Tween 20, a non-ionic surfactant, is well-known for its ability to enhance enzymatic hydrolysis of lignocellulosic biomass (Seo et al., 2011; Chen et al., 2018; Wang et al., 2020). In this study, Tween 20 supplementation into co-cultures of *C. thermocellum* and *T. celere* strain A9 was investigated to assess the impact on saccharification of alkaline-treated rice straw. Various concentrations of Tween 20 (0.1 to 1.5% v/v) were tested with a substrate loading of 150 g/l and a 2-day culture of *C. thermocellum* inoculated with *T. celere* strain A9. The highest glucose production, 90.8 g/l, was observed at 0.5% (v/v) Tween 20, corresponding to 75.7% of the theoretical glucose yield. This yield was significantly higher than the control (78.8 g/l or 65.7% of the theoretical glucose yield) and other concentrations (0.1, 1.0, and 1.5% v/v) (Table 2). Compared to control, glucose production increased by 10.17% at 0.5% (v/v) Tween 20, however, the highest concentration (1.5% v/v) led to a 19.8% decrease in glucose yield, suggesting that excessive Tween 20 hinders saccharification in the co-culture system. These results indicate that a concentration of 0.5% (v/v) Tween 20 is optimal for saccharification of treated rice straw in this co-culture system (Figure 6). Statistical analysis provided in Table 2 confirms significant differences in glucose yields across the tested Tween 20 concentrations.

**Table 2:** Effect of Tween 20 supplementation on the saccharification of treated rice straw at a solid loading of 150 g/l using a co-culture of *C. thermocellum* and *T. celere* strain *A9.*

Tween 20 (% v/v)	Accumulated glucose concentration (g/l)	Glucose yield* (%)	Cellulase (U/ml)	β-Glucosidase (U/ml)
–	78.83 ± 0.15^b^	65.69 ± 0.15^b^	2.54 ± 0.28^c^	3.01 ± 0.18^c^
0.1	82.52 ± 0.25^b^	68.77 ± 0.25^b^	3.28 ± 0.17^b^	4.45 ± 0.14^b^
0.5	90.84 ± 0.23^a^	75.70 ± 0.23^a^	5.15 ± 0.12^a^	5.25 ± 0.24^a^
1.0	80.83 ± 0.53^b^	67.36 ± 0.53^b^	2.84 ± 0.34^bc^	4.51 ± 0.27^b^
1.5	54.83 ± 0.47^c^	45.28 ± 0.47^c^	1.32 ± 0.32^d^	1.12 ± 0.11^d^

The accumulated glucose concentration, glucose yield, and retained activities of cellulase and β-glucosidase were measured after 7 days of culture with Tween 20 concentrations of 0.1, 0.5, 1.0, and 1.5% (v/v), as well as a control without Tween 20. Values are the means of triplicate experiments ± SD. Different uppercase letters indicate significant differences among various concentration of Tween 20 (Tukey test, *p* < 0.05). ^*^Glucose yield (%) was calculated based on the amount of glucose released relative to the dry weight of treated rice straw.

After the completion of saccharification in the co-culture system, retained cellulase and *β*-glucosidase activities were measured. Without Tween 20, the retained cellulase and β-glucosidase activities were 2.54 U/ml and 3.01 U/ml, respectively. With Tween 20 supplementation, the highest cellulase and β-glucosidase activities (5.15 U/ml and 5.25 U/ml) were observed at 0.5% (v/v), which were significantly higher than at other concentrations (Table 2). These results indicate that 0.5% (v/v) Tween 20 not only enhances glucose production but also improves enzyme retention in the co-culture system, making it the optimal concentration for subsequent experiments.

### 3.3 Semi-fed-batch saccharification of treated rice straw using co-culture and Tween 20

Upon saccharification of alkaline-treated rice straw in a co-culture of *C. thermocellum* and *T. celere* strain A9 with 150 g/l solid loading and 0.5% (v/v) Tween 20 supplementation, significant levels of cellulase and β-glucosidase activity was detected in the culture medium. To exploit this residual enzymatic activity, a semi-fed-batch approach was employed by introducing additional substrate (i.e., alkaline-treated rice straw) into the culture.

**Figure 7:**
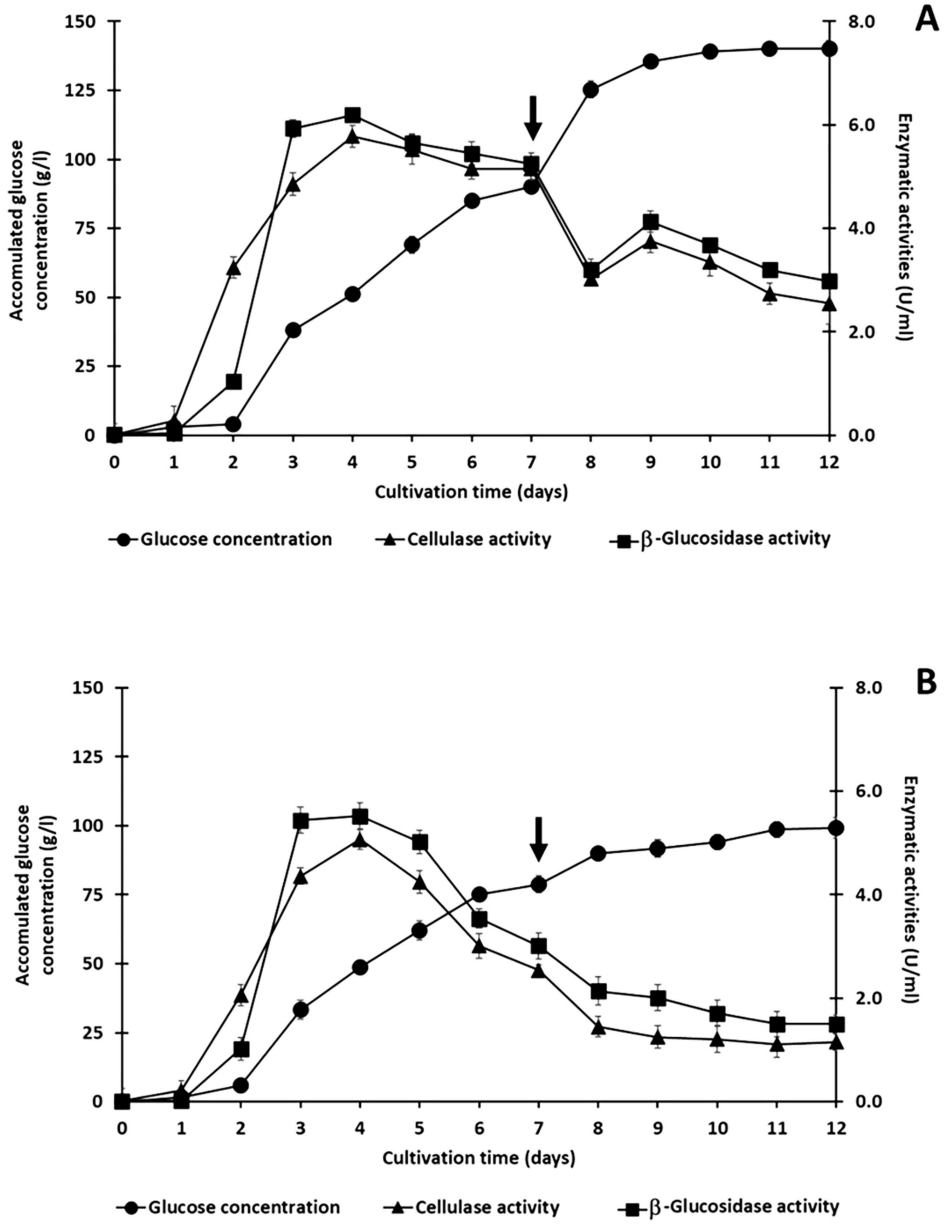
Semi-fed-batch saccharification of treated rice straw using a co-culture of *C. thermocellum* and *T. celere* strain A9. The process was initiated with an initial solid loading of 150 g/l, followed by incremental additions of 100 g/l of treated rice straw, supplemented with 0.5% (v/v) Tween 20 **(A)** and without Tween 20 **(B)**. The arrow indicates the time point of substrate addition. Data represent the means of three independent experiments, with error bars indicating the standard deviation (*n* = 3).

In the initial saccharification phase, the co-culture system produced 90.8 g/l of glucose from the 150 g/l treated rice straw. The retained cellulase and β-glucosidase activities were 5.15 U/ml and 5.25 U/ml, respectively (Figure 7A). After the initial phase, another 100 g/l of alkaline-treated rice straw was introduced into the culture on day 7. This also resulted in a significant increase in glucose production. An additional 49.2 g/l of glucose was produced, leading to a total glucose concentration of 140 g/l from the 250 g/l treated rice straw loading. This is a glucose yield of 70.1%. Interestingly, the retained cellulase and β-glucosidase activities decreased to 2.55 U/ml and 2.98 U/ml, respectively, after this second loading (Figure 7A).

In contrast, co-culture without Tween 20 supplementation produced only 78.83 g/l of glucose from the initial 150 g/l of treated rice straw, which is significantly lower than the glucose concentration achieved with Tween 20 (Figure 7B). After the addition of 100 g/l of alkaline-treated rice straw as a second load, the glucose concentration increased to 99.24 g/l.

The residual enzyme activities after the first phase were 2.54 U/ml for cellulase and, 3.01 U/ml for β-glucosidase. These are significantly lower than the corresponding activities in cultures supplemented with Tween 20. Following the second phase, enzyme activities further declined to 1.50 U/ml and 1.15 U/ml for *β*-glucosidase and cellulase, respectively (Figure 7B).

Results indicate that semi-fed-batch saccharification via co-culture of *C. thermocellum* and *T. celere* strain A9, supplemented with Tween 20, significantly enhances glucose production and enzymatic activity during the saccharification of alkaline-treated rice straw at high solid loading. Notably, when comparing this method to traditional batch saccharification, which uses 250 g/l of alkaline-treated rice straw as the initial substrate, only 55.5 g/l of glucose production with a theoretical yield of 27.7% is achieved (Figure 4).

### 3.4 Fermentation of glucose via semi-fed-batch co-culture saccharification to ethanol

**Figure 8:**
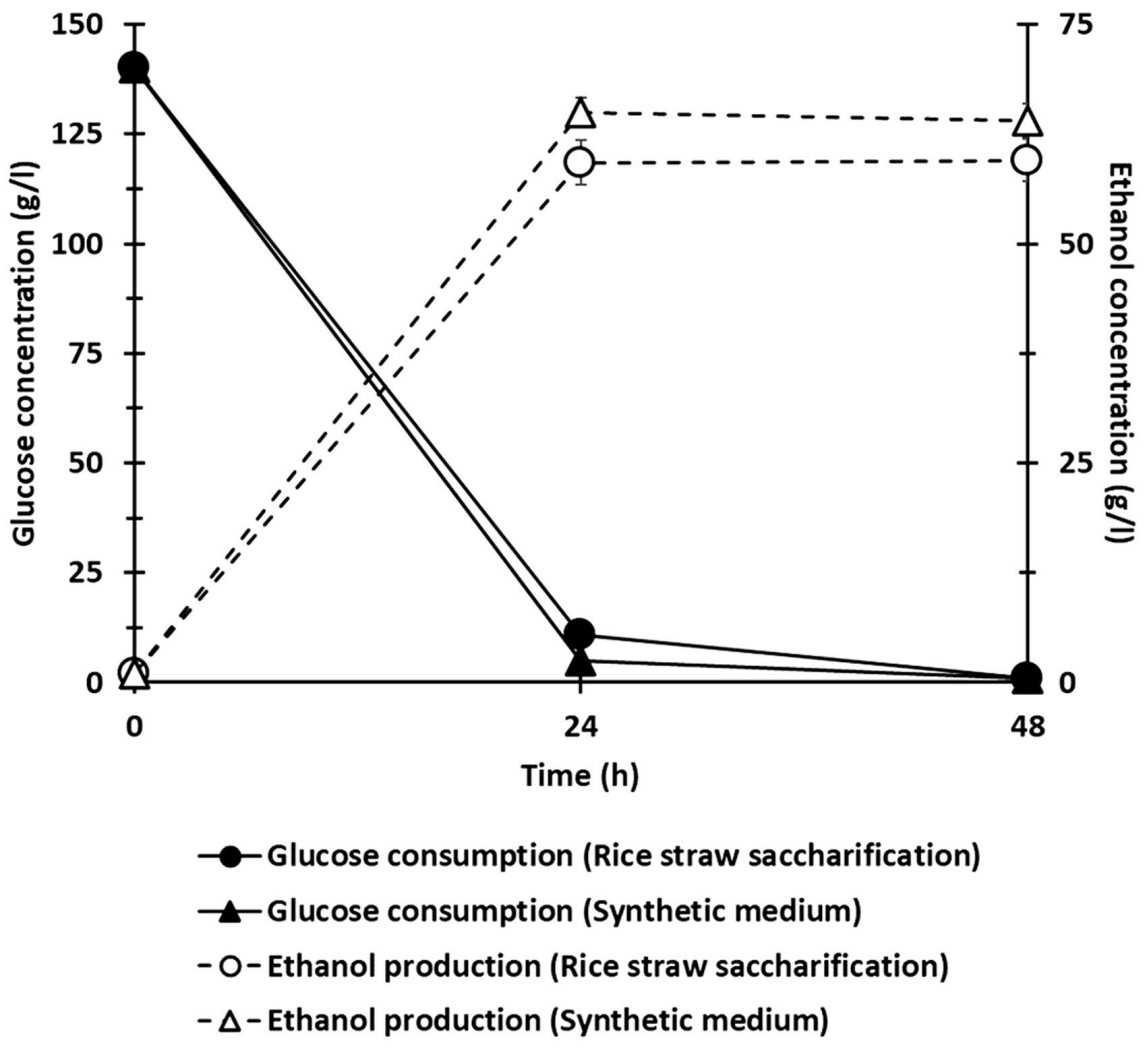
Ethanol fermentation profile using saccharification slurry from the co-culture *of C. thermocellum* and *T. celere* strain A9. Error bars represent the standard deviation (*n* = 3).

To assess the feasibility of converting glucose derived from the semi-fed-batch saccharification of treated rice straw into ethanol, *S. cerevisiae* was directly inoculated into the slurry obtained from the co-culture of *C. thermocellum* and *T. celere* strain A9 without the addition of any extra nutrients. The slurry contained a glucose concentration of 140.0 g/l and a small amount of cellobiose at 4.5 g/l. Acetic acid, lactic acid, and ethanol were not detected due to their very low concentrations. Fermentation profiles for ethanol production and glucose consumption were obtained from the slurry (Figure 8), along with profiles from a reference fermentation test using a medium containing the same glucose concentration. The fermentation of the co-culture slurry with *S. cerevisiae* resulted in an ethanol concentration of 59.2 g/l, which corresponds to approximately 75.9% of the theoretical maximum yield. Results suggest that the semi-fed-batch saccharification slurry from the *C. thermocellum* and *T. celere* strain A9 co-culture system can be directly fermented to ethanol by *S. cerevisiae* without any inhibitory effects. The high ethanol yield indicates the potential of this approach for efficient bioethanol production from lignocellulosic biomass.

## 4 Discussion

Cost-effective production of bio-based chemicals and fuels from lignocellulosic biomass through enzymatic processes remains a significant challenge and motivates research. A major challenge in hydrolyzing lignocellulose is maximizing cellulolytic enzyme efficiency.

Use of microbial cultures for saccharifying lignocellulosic substrate is a promising approach (Taha et al., 2015; Du et al., 2020; Beri et al., 2020; Akdemir et al., 2022; Zou et al., 2023).

*C. thermocellum* is renowned as one of the most effective microorganisms for degrading lignocellulosic biomass. Indeed, *C. thermocellum* shows a 2- to 4-fold greater effectiveness than commercial fungal cellulases in solubilizing woody and herbaceous lignocellulosic feedstocks (Lynd et al., 2017; Holwerda et al., 2019). The thermophilic nature of *C. thermocellum* further enhances its suitability for industrial biomass conversion by reducing contamination risks at higher culture temperatures while accelerating reaction rates (Lynd et al., 2002; Akinosho et al., 2014; Beri et al., 2020; Du et al., 2020; Mazzoli and Olson, 2020). However, *C. thermocellum*’s drawback is that it does not express extracellular *β*-glucosidases. This induces a feedback inhibition against its cellulosome via cellobiose accumulation. *T. celere* strain A9, which produces extracellular β-glucosidase, serves as an ideal co-culture partner that will hydrolyze cellobiose into glucose, thereby mitigating this feedback inhibition. Moreover, *T. celere* strain A9 is compatible with the growth conditions of *C. thermocellum*, ensuring a stable and efficient co-culture system (Nhim et al., 2022).

This study investigated the saccharification potential of various agricultural wastes including corn hull, oil palm empty fruit bunches, rice straw, and sugarcane bagasse using a co-culture of *C. thermocellum* and *T. celere* strain A9. These feedstocks were alkaline-treated, which has been shown to be effective in enhancing the saccharification efficiency of biomass (Liu et al., 2018; Das et al., 2021). Indeed, alkaline-treated rice straw exhibits saccharification efficiency comparable to that obtained from microcrystalline cellulose when co-cultured with *C. thermocellum* and *T. celere* strain A9 (Nhim et al., 2022). The high conversion efficiency of treated rice straw is facilitated by its high cellulose and low lignin contents. Therefore, alkaline-treated rice straw was chosen as the model lignocellulosic biomass for this study.

### 4.1 Synergy and cooperation between *C. thermocellum* and *T. celere* strain A9

Synergistic interactions between *C. thermocellum* and *T. celere* strain A9 play a pivotal role in obtaining high glucose concentrations from alkaline-treated rice straw. Both strains exhibit growth compatibility under identical culture conditions. Whereas *C. thermocellum* produces cellulosomes, which effectively hydrolyze cellulose into cello-oligosaccharides and cellobiose, *T. celere* strain A9 expresses extracellular β-glucosidase that reduces cellobiose to glucose—a function lacking in *C. thermocellum*.

Interestingly, the timing of *T. celere* strain A9 inoculation into *C. thermocellum* cultures significantly impacts glucose production levels (see Figure 3). Our data indicate that pre-cultivation of *C. thermocellum* for 1–2 days before inoculating *T. celere* strain A9 results in higher glucose concentrations with no notable increase in efficiency for pre-cultivation times great than 2 days. This result is likely due to cellulosome component expression and assembly during the first couple of days of culture after which time the cellulosomes are saturated with substrate and cellobiose has accumulated thus inducing feedback inhibition.

*T. celere* strain A9 proliferates rapidly in culture, particularly those rich in cellobiose. Moreover, *T. celere* strain A9 produces extracellular β-glucosidases that efficiently hydrolyze cellobiose. When inoculated into *C. thermocellum* cultures featuring cellobiose accumulation, *T. celere* strain A9 mitigates feedback inhibition on *C. thermocellum* cellulosomes. This enhances overall cellulose hydrolysis efficiency and increases glucose production.

*C. thermocellum* expresses a high-affinity uptake system for cellodextrins that parallels cellulose hydrolysis by cellulosomes. However, affinity for glucose uptake is low compared to cellobiose uptake, which explains the observed high glucose accumulation. It also explains why *C. thermocellum* has a long lag phase when growing on glucose alone (Strobel et al., 1995; Ha-Tran et al., 2021). Low-affinity glucose uptake in *C. thermocellum* in combination with the extracellular β-glucosidase activity of *T. celere* strain A9 results in high glucose production efficiency.

The synergy between *C. thermocellum* and *T. celere* strain A9 is highly adaptable to different lignocellulosic substrates. The alkaline pretreatment used in this study has been demonstrated to enhance the saccharification efficiency of various agricultural residues by improving cellulose accessibility and reducing lignin interference (Liu et al., 2018). Although this study focused on rice straw, the co-culture’s synergistic can be optimized for other lignocellulosic materials, such as corn hulls, oil palm empty fruit bunches, and sugarcane bagasse, each of which differs in structure and contains varying amounts of cellulose, hemicellulose, and lignin. By adjusting pretreatment conditions, inoculation timing and solid loading, the co-culture system can be tailored to maximize saccharification efficiency across a wide range of feedstocks.

### 4.2 *C. thermocellum* and *T. celere* A9 co-culture and high solids loading

Solids loading is an important factor impacting enzymatic hydrolysis efficiency in the breakdown of lignocellulosic biomass. Indeed, limitations in solids loading impacts glucose production efficiency and the overall economic feasibility of bioconversion processes. Operating at higher solid loadings can increase product yields, reduce water usage, and enhance reactor efficiency. Methods that are efficient under high solids loading are attractive for industrial applications. This study evaluated the efficiency and optimal substrate loading for maximizing glucose production using the co-culture of *C. thermocellum* and *T. celere* strain A9. The highest glucose concentration was achieved at 150 g/l substrate loading. However, at loadings above 150 g/l, both glucose concentration and yield decreased, particularly at 250 g/l. At higher substrate concentrations, factors such as inefficient mixing, increased viscosity, limited growth of the bacteria, and reduced enzyme production capacity likely contributed to decreased glucose production and reduced saccharification efficiency (Modenbach and Nokes, 2013; Jung et al., 2015; da Silva et al., 2020; Agrawal et al., 2021; Shiva et al., 2022). These findings highlight the effectiveness of the co-culture system, particularly at a substrate loading of 150 g/l, and its potential for industrial applications requiring high substrate concentrations.

### 4.3 Tween 20 enhances glucose yields in *C. thermocellum* and *T. celere* strain A9 co-culture

The selection of Tween 20 over alternative surfactants for this research is based on its mild, non-ionic nature, which prevents enzyme denaturation and ensures effective interaction with lignocellulose (Chen et al., 2018). Additionally, its compatibility with microbial consortia makes it a promising additive for optimizing bioconversion processes (Eckard et al., 2013; Cui et al., 2023). Regarding cost, Tween 20 is generally considered an affordable surfactant, making it a cost-effective option for large-scale industrial applications.

In the enzymatic saccharification of lignocellulosic biomass, lignin tends to non-specifically adsorb enzymes, reducing their efficiency. However, the adsorption of Tween 20 onto lignin prevents the unproductive binding of enzymes. As a result, Tween 20 enhances saccharification efficiency by mitigating nonproductive enzyme binding to lignin, increasing enzyme activity, improving substrate accessibility, and promoting enzyme cooperation (Eriksson et al., 2002; Eckard et al., 2013; Cui et al., 2023). Numerous studies have shown that Tween 20 can effectively enhance the enzymatic hydrolysis of lignocellulosic biomass (Seo et al., 2011; Liu et al., 2016; Chen et al., 2018; Wang et al., 2020; Cui et al., 2023).

Addition of Tween 20 (0.1 to 1.0% v/v) in this study significantly improved glucose yields compared to controls, underscoring its positive impact on saccharification efficiency. Furthermore, Tween 20 appears to help maintain both cellulase and *β*-glucosidase activities in the co-culture system post-saccharification. However, at higher Tween 20 concentrations (1.5% v/v), we observed the inhibitory effects, emphasizing the need for careful optimization of Tween 20 dosage. Excessive concentrations can disrupt microbial interactions and enzymatic functions, leading to reduced saccharification efficiency (Muñoz et al., 2022).

### 4.4 Semi-fed-batch method in *C. thermocellum* and *T. celere* strain A9 co-culture enhance glucose yields

The semi-fed-batch method for substrate delivery enhances saccharification efficiency by effectively using residual enzyme activity in *C. thermocellum* and *T. celere* strain A9 co-cultures supplemented with Tween 20 to maximize glucose yields. This two-stage substrate delivery strategy increased glucose production to 140 g/l, a 70.1% yield, from a total of 250 g/l of treated rice straw (150 g/l and 100 g/l substrate loading). In contrast, simple batch saccharification using 250 g/l of treated rice straw as the initial substrate load resulted in a glucose concentration of 55.5 g/l, a glucose yield of only 27.7%. High substrate loading in a single batch can lead to inefficient mixing and low water activity, negatively impacting the bacterial growth and saccharification efficiency (Jung et al., 2015; da Silva et al., 2020). By staging substrate loading into two phases, better mixing and adequate water activity are realized, thereby enhancing the overall efficiency of the process. This study represents the first report of semi-fed-batch saccharification with high lignocellulose loads using *C. thermocellum* and *T. celere* strain A9 co-culture. Our data highlight the potential of this method for improving saccharification efficiency and reducing the need for external enzymes. By mitigating the risks associated with high single-time substrate loading, this method provides a practical and efficient solution for managing high solids loading in industrial saccharification processes.

The rapid decrease in enzyme activity after adding fresh alkaline-treated rice straw to co-culture (see Figure 7A) suggests significant enzyme binding to the newly added substrate. Robust substrate binding is a major factor underlying rapid increases in glucose concentration. The slower saccharification rate observed in the first substrate loading compared to the second is attributed to lag phase kinetics associated with bacterial growth and enzyme expression.

During the second substrate loading, bacterial cultures are well within late log phase to stationary phase in which peak growth and enzyme expression is at a maximum. Thus, the co-culture system is primed for hydrolyzing newly added substrate using resident enzymes. Despite increased saccharification rates during the second substrate loading, yield was only 61.7%, which is notably lower than the 75.7% yield achieved in the first substrate loading. The decrease in yield may be attributed to enzyme inhibition caused by glucose accumulation, a loss of enzyme stability over time, or both. The gradual decrease in enzyme activity during the second stage may be caused by several factors. For example, enzymes may bind to lignin or other residual materials, leading to reduced hydrolytic activity on target substrates. Additionally, high glucose concentrations may induce feedback inhibition on key enzymes. In the absence of Tween 20 (Figure 7B), enzyme binding to lignin likely contributes to the observed lower enzyme activity and glucose yield, as such interactions limit the availability of enzymes for hydrolysis of target molecules. Our results underscore the crucial role that Tween 20 plays in mitigating non-productive enzyme binding to lignin and other residues within the substrate. Moreover, Tween 20 reduces surface tension, enhances enzyme accessibility, and improves overall saccharification efficiency under high solid loading conditions.

In this study, the co-culture of *C. thermocellum* and *T. celere* strain A9 achieved a glucose concentration of 140 g/l from 250 g/l of alkaline-treated rice straw (approximately 200 g/l glucan), corresponding to 70.1% of the theoretical glucose yield without the addition of external enzymes. This yield is comparable to or higher than those previously reported for conventional enzymatic hydrolysis methods. For instance, enzymatic hydrolysis of ammonia-treated rice straw using *C. thermocellum* cellulosome and *Thermoanaerobacter brockii β*-glucosidase achieved 91% glucan hydrolysis. However, this process required external enzyme supplementation and was conducted at a significantly lower substrate loading of 10 g/l glucan-loaded treated rice straw (Waeonukul et al., 2012). Similarly, enzymatic hydrolysis of 30 g/l glucan-loaded treated rice straw using commercial enzymes (15 FPU of Celluclast 1.5 L and 30 CBU of β-glucosidase from Novozyme 188) achieved 71.1% of the theoretical glucose yield (Ko et al., 2009). Optimized enzymatic hydrolysis of alkaline-treated rice straw using a combination of multiple commercial enzymes (Cellulase Onozuka 3S, Cellulase T Amano 4, and Pectinase G Amano) resulted in a glucose concentration of 75.3 g/l with a hydrolysis efficiency of 94.1% at 100 g/l rice straw loading (Takano and Hoshino, 2018); however, this approach required extensive optimization and relied on costly commercial enzyme formulations. In another study, *C. thermocellum* cultures supplemented with thermostable β-glucosidase yielded glucose concentrations of 80.4 g/l, achieving a 72% glucan conversion at 100 g/l glucan from alkaline-treated rice straw loading (Prawitwong et al., 2013). Therefore, the semi-fed-batch saccharification method using the co-culture of *C. thermocellum* and *T. celere* strain A9 in this study not only achieved similar or higher glucose yields but also processed significantly higher solid loadings, eliminating the need for commercial enzymes through *in situ* enzyme production. These results highlight the advantages of the co-culture approach, including its scalability and cost-effectiveness, making it a promising candidate for industrial biofuel production.

The co-culture system of *C. thermocellum* and *T. celere* strain A9 shows significant feasibility for industrial-scale saccharification, although some challenges must be considered for scalability. One key limitation is maintaining stable co-cultures at large scale, which requires precise control of environmental conditions such as temperature, pH, nutrient levels, and anaerobic conditions, potentially increasing operational costs. However, with appropriate bioreactor design and equipment, these challenges can be managed. Additionally, the reduction in reliance on commercial enzymes could offset some of the increased costs, enhancing overall cost-effectiveness.

For industrial applications, further optimization is needed to accommodate higher solids loadings, which are often required in commercial biofuel production. This could be achieved through refined feeding strategies, such as gradual biomass addition, to prevent inhibition effects and maintain optimal metabolic activity. Improving mixing is crucial for enhancing nutrient and gas transfer, ensuring even substrate availability throughout the reactor, which supports bacterial growth, as well as simultaneous enzyme production and saccharification. Additionally, optimizing additives like Tween 20 to reduce viscosity and improve enzyme accessibility would further enhance the system’s performance at higher solids loadings, making it a promising solution for industrial biofuel production.

### 4.5 Slurry from *C. thermocellum* and *T. celere* strain A9 co-culture supports fermentation

Successful fermentation of glucose to ethanol from the semi-fed-batch slurry derived from the *C. thermocellum* and *T. celere* strain A9 co-culture system was demonstrated (Figure 8). Using *S. cerevisiae* to directly convert glucose-rich *C. thermocellum* and *T. celere* strain A9 co-culture slurry, an ethanol concentration of 59.2 g/l was achieved, corresponding to a 75.9% theoretical yield, which is considered high conversion efficiency. Notably, no additional nutrients were added to the fermentation medium suggesting that additional optimization may produce even higher yields. Also notable was the absence of any inhibitory effects during fermentation. This is advantageous since many lignocellulosic saccharification processes produce by-products such as acetic acid, lactic acid, or furfural that can inhibit microbial fermentation, leading to lower bioethanol yields (Palmqvist et al., 1999; Yuan et al., 2017). The minimal presence of these inhibitors in our slurry suggests that the *C. thermocellum* and *T. celere* strain A9 co-culture system not only efficiently hydrolyzes the biomass but also produces a clean substrate for subsequent yeast-mediated fermentation.

### 4.6 Advantage of the semi-fed-batch method in *C. thermocellum* and *T. celere* strain A9 co-culture

The semi-fed-batch saccharification method developed in this study represents a significant advancement in lignocellulosic biomass conversion by offering a distinct approach from consolidated bioprocessing (CBP) strategies, which integrate enzyme production, biomass hydrolysis, and fermentation into a single step. For example, CBP may utilize a microorganism such as *C. thermocellum* to perform simultaneous lignocellulose breakdown and glucose fermentation to ethanol without requiring exogenous enzyme supplementation (Lynd et al., 2005; Akinosho et al., 2014; Lynd et al., 2017; Beri et al., 2020; Du et al., 2020). While CBP is recognized as a promising, cost-effective solution for efficient biomass conversion, there are multiple limitations. Such limitations have restricted CBP application in large-scale ethanol production.

A major limitation of *C. thermocellum*-based CBP is relatively low ethanol yields and poor ethanol tolerance (Pang et al., 2018; Olson et al., 2023), which prevents high ethanol concentrations necessary for commercially viable biofuel production. Another drawback of CBP is limits on substrate loading. High substrate loading often leads to inefficient mixing and reduced enzyme accessibility to target molecules at high solids loading. CBP is also susceptible to feedback inhibition resulting in deactivation or non-productive binding of system enzymes to non-target molecules (e.g., lignin).

In contrast, our semi-fed-batch strategy mitigates the afore-mentioned CBP limitations by introducing substrate in stages, which enhances substrate conversion efficiency, reduces enzyme inhibition, and produces a glucose-rich slurry amenable to efficient yeast-based ethanol fermentation processes. The semi-fed-batch method not only introduces substrate in two stages for optimal glucose production but also separates enzymatic hydrolysis from the fermentation process, enabling separate optimization of saccharification and fermentation.

## 5 Conclusion

In conclusion, this study suggests that a semi-fed-batch saccharification approach using a *C. thermocellum* and *T. celere* strain A9 co-culture supplemented with Tween 20 is an effective strategy for enhancing the enzymatic conversion of lignocellulosic biomass even under high solids loading conditions. The strategic timing of *T. celere* strain A9 inoculation into pre-cultures of *C. thermocellum*, the use of Tween 20, and optimization of solid loading, significantly improves glucose production efficiency. This method not only enhances enzymatic saccharification but also dependence on costly external enzyme supplements, providing a cost-effective and scalable solution for biomass conversion. By addressing key challenges in lignocellulosic biomass bioconversion, this approach offers great potential to advance sustainable biofuel production tailored to industrial applications.

## Data Availability

The original contributions presented in the study are included in the article/Supplementary material, further inquiries can be directed to the corresponding authors.

## Footnotes

^1^ http://www.nrel.gov/biomass/analytical_procedures.html
