# Supplementary material for: Effective semi-fed-batch saccharification with high lignocellulose loading using co-culture of Clostridium thermocellum and Thermobrachium celere strain A9
Source: Front Microbiol. 2025 Jan 7;15:1519060. doi: 10.3389/fmicb.2024.1519060 (PMC11747163; doi:10.3389/fmicb.2024.1519060)
Supplement: Supplementary file 1 [file Table_1.docx]

**Supplementary materials**

**Table S1** Effect of inoculation timing of *T. celere* strain A9 on the saccharification of treated rice straw at a solid loading of 50 g/l using co-culture with *C. thermocellum*. Values are the means of triplicate experiments ± SD.

| Cultivation time (days) | Accumulated glucose concentration (g/l) | | | | |
| --- | --- | --- | --- | --- | --- |
|  | *C. thermocellum* only | *C. thermocellum*  and *T. celere* A9 inoculated simultaneously | *C. thermocellum* culture prior to  *T. celere* A9 inoculation for  1 day | *C. thermocellum* culture prior to  *T. celere* A9 inoculation for  2 days | *C. thermocellum* culture prior to  *T. celere* A9 inoculation for  3 days |
| 0 | 0.02 ± 0.00^a^ | 0.01 ± 0.00^a^ | 0.05 ± 0.00^a^ | 0.02 ± 0.00^a^ | 0.02 ± 0.00^a^ |
| 1 | 0.22 ± 0.05^c^ | 3.21 ± 0.02^a^ | 0.63 ± 0.01^b^ | 0.72 ± 0.04^b^ | 0.67 ± 0.04^b^ |
| 2 | 3.15 ± 0.05^b^ | 6.01 ± 0.01^a^ | 5.68 ± 0.45^a^ | 3.25 ± 0.54^b^ | 2.88 ± 0.10^b^ |
| 3 | 5.95 ± 0.05^d^ | 8.16 ± 0.05^c^ | 9.61 ± 0.57^b^ | 12.27 ± 0.63^a^ | 5.38 ± 0.58^d^ |
| 4 | 6.73 ± 0.08^d^ | 12.55 ± 0.12^c^ | 14.08 ± 0.56^b^ | 19.45 ± 0.04^a^ | 13.69 ± 0.14^b^ |
| 5 | 7.24 ± 0.12^d^ | 15.47 ± 0.15^c^ | 19.89 ± 0.56^b^ | 24.91 ± 0.54^a^ | 24.04 ± 0.13^a^ |
| 6 | 8.16 ± 0.52^d^ | 18.55 ± 0.22^c^ | 22.34 ± 0.55^b^ | 28.02 ± 0.43^a^ | 27.72 ± 0.14^a^ |
| 7 | 8.23 ± 0.15^d^ | 18.94 ± 0.28^c^ | 23.01 ± 0.48^b^ | 28.81 ± 0.54^a^ | 28.80 ± 0.63^a^ |

Different uppercase letters indicate significant differences among various of inoculation timing of bacterial strain on glucose product (Tukey test, *p* < 0.05).

**Table S2** Effect of solid loading (50 to 250 g/l) on the saccharification of treated rice straw using a co-culture of *C. thermocellum* and *T. celere* strain A9. Values are the means of triplicate experiments ± SD.

| Cultivation time (days) | Accumulated glucose concentration (g/l) | | | | |
| --- | --- | --- | --- | --- | --- |
|  | 50 g/l  Solid loading | 100 g/l  Solid loading | 150 g/l  Solid loading | 200 g/l  Solid loading | 250 g/l  Solid loading |
| 0 | 0.02 ± 0.00^a^ | 0.02 ± 0.00^a^ | 0.02 ± 0.00^a^ | 0.09 ± 0.00^a^ | 0.01 ± 0.00^a^ |
| 1 | 0.67 ± 0.22^b^ | 0.67 ± 0.57^b^ | 1.67 ± 1.25^ab^ | 3.67 ± 1.57^a^ | 1.55 ± 0.10^ab^ |
| 2 | 0.88 ± 1.20^b^ | 0.88 ± 1.30^b^ | 5.88 ± 1.20^a^ | 8.89 ± 1.40^a^ | 5.62 ± 0.50^a^ |
| 3 | 8.38 ± 1.30^d^ | 18.38 ± 1.40^c^ | 33.38 ± 3.50^a^ | 26.39 ± 2.40^b^ | 21.15 ± 0.88^c^ |
| 4 | 14.68 ± 2.20^c^ | 27.68 ± 2.30^b^ | 48.68 ± 2.35^a^ | 43.68 ± 1.26^a^ | 31.69 ± 1.35^b^ |
| 5 | 23.03 ± 1.17^d^ | 39.03 ± 1.26^c^ | 62.03 ± 3.50^a^ | 54.03 ± 2.46^b^ | 42.04 ± 1.26^c^ |
| 6 | 27.24 ± 1.20^d^ | 53.24 ± 2.30^c^ | 75.24 ± 2.30^a^ | 63.23 ± 1.40^b^ | 53.48 ± 0.73^c^ |
| 7 | 28.88 ± 2.18^d^ | 56.19 ± 2.49^c^ | 78.83 ± 2.78^a^ | 65.33 ± 2.51^b^ | 55.48 ± 1.16^c^ |

Different uppercase letters indicate significant differences among different of solid loading on the saccharification of treated rice straw (Tukey test, *p* < 0.05).
